# Effect of Contract Compliance Rate to a Fourth-Generation Telehealth Program on the Risk of Hospitalization in Patients With Chronic Kidney Disease: Retrospective Cohort Study

**DOI:** 10.2196/jmir.8914

**Published:** 2018-01-24

**Authors:** Chi-Sheng Hung, Jenkuang Lee, Ying-Hsien Chen, Ching-Chang Huang, Vin-Cent Wu, Hui-Wen Wu, Pao-Yu Chuang, Yi-Lwun Ho

**Affiliations:** ^1^ Telehealth Center, National Taiwan University Hospital Taipei Taiwan; ^2^ Department of Internal Medicine, National Taiwan University Hospital Taipei Taiwan

**Keywords:** telehealth, contract compliance rate, chronic kidney disease

## Abstract

**Background:**

Chronic kidney disease (CKD) is prevalent in Taiwan and it is associated with high all-cause mortality. We have shown in a previous paper that a fourth-generation telehealth program is associated with lower all-cause mortality compared to usual care with a hazard ratio of 0.866 (95% CI 0.837-0.896).

**Objective:**

This study aimed to evaluate the effect of renal function status on hospitalization among patients receiving this program and to evaluate the relationship between contract compliance rate to the program and risk of hospitalization in patients with CKD.

**Methods:**

We retrospectively analyzed 715 patients receiving the telehealth care program. Contract compliance rate was defined as the percentage of days covered by the telehealth service before hospitalization. Patients were stratified into three groups according to renal function status: (1) normal renal function, (2) CKD, or (3) end-stage renal disease (ESRD) and on maintenance dialysis. The outcome measurements were first cardiovascular and all-cause hospitalizations. The association between contract compliance rate, renal function status, and hospitalization risk was analyzed with a Cox proportional hazards model with time-dependent covariates.

**Results:**

The median follow-up duration was 694 days (IQR 338-1163). Contract compliance rate had a triphasic relationship with cardiovascular and all-cause hospitalizations. Patients with low or very high contract compliance rates were associated with a higher risk of hospitalization. Patients with CKD or ESRD were also associated with a higher risk of hospitalization. Moreover, we observed a significant interaction between the effects of renal function status and contract compliance rate on the risk of hospitalization: patients with ESRD, who were on dialysis, had an increased risk of hospitalization at a lower contract compliance rate, compared with patients with normal renal function or CKD.

**Conclusions:**

Our study showed that there was a triphasic relationship between contract compliance rate to the telehealth program and risk of hospitalization. Renal function status was associated with risk of hospitalization among these patients, and there was a significant interaction with contract compliance rate.

## Introduction

Telehealth has been increasingly used in the management of chronic conditions such as diabetes, asthma, and cardiovascular disease (CVD) [1-4]. A fourth-generation telehealth program is an Internet-based, synchronized disease management program using telemonitoring technology to provide an immediate response. In a prior report, we have shown that 576 patients with chronic CVD who received a fourth-generation telehealth care program had lower mortality compared to 1178 patients who received usual care [5]. The adjusted hazard ratio of all-cause mortality for the use of telehealth was 0.866 (95% CI 0.837-0.896). Based on these results, this technology has been increasingly applied to the care of other chronic conditions in Taiwan.

Chronic kidney disease (CKD) is a chronic condition characterized by a decreased glomerular filtration rate (GFR; <60 mL/min/1.73 m^2^) and associated with risks of progressive deterioration in kidney function and of CVD [6]. To halt the deterioration in renal function, the Kidney Disease: Improving Global Outcomes organization published CKD management guidelines, which recommend treatment of the causes of the disease and of factors that may aggravate kidney deterioration [7]. Compliance to the guidelines and recommended treatments is crucial for the long-term management of patients with CKD [8]. Telehealth programs have potential advantages in ensuring treatment compliance and early recognition of disease complications. Telehealth initiatives in which nephrology specialists provide their expertise remotely over the Internet represent a model that can be adopted in low-resource settings [9]. There have been a few studies on the use of telemedicine among patients receiving dialysis [10,11] or renal transplant recipients [12], but none on the use of telehealth programs for the management of patients with stage 3 to 4 CKD.

Chronic kidney disease is prevalent in Taiwan and is associated with high all-cause mortality [13]. We have shown that a fourth-generation telehealth program is associated with lower mortality compared with usual care [5]. However, the influence of renal function status on hospitalization among patients receiving the program has not previously been evaluated. Similarly, the relationship between contract compliance rate to the program and risk of hospitalization in patients with CKD has not yet been reported. The aim of this study was to elucidate if the contract compliance rate to the telehealth program is associated with a higher rate of hospitalization among patients with or without CKD, and to identify any possible interactions between contract compliance rate and CKD. We designed this retrospective cohort study to answer this question.

## Methods

### Study Design

This was a single-center, retrospective study that was approved by the Institutional Review Board of National Taiwan University Hospital, Taipei, Taiwan. Informed consent was obtained from all participants.

### Recruitment

The study was conducted from December 2009 to April 2013 at the Telehealth Center of the hospital by the Taiwan ELEctroHEALTH Study Group (TELEHEALTH Study Group). Patients older than 20 years with diagnosed chronic CVD and receiving the telehealth program at our telehealth center were enrolled as the study group. The decision of whether to receive the telehealth program depended on the patients and/or their caregivers. Chronic CVD included coronary artery disease, myocardial infarction, heart failure, peripheral artery disease, stroke, and hypertension.

### Definition of Chronic Kidney Disease

The definition of CKD is based on the Kidney Disease Outcomes Quality Initiative guidelines published by the National Kidney Foundation [14]. Chronic kidney disease is defined as a GFR of less than 60 mL/min/1.73 m^2^. The renal function status is classified into (1) normal: no kidney damage, with a GFR ≥60 mL/min/1.73 m^2^; (2) CKD: kidney damage, with GFR <60 mL/min/1.73 m^2^; or (3) end-stage renal disease (ESRD): patient on maintenance dialysis, including hemodialysis and peritoneal dialysis.

### Telehealth Program

The fourth-generation telehealth program at our telehealth center is a synchronized and integrated remote management program for chronic conditions. The Internet-based platform was developed by the Graduate Institute of Biomedical Electronics and Bioinformatics, National Taiwan University, Taiwan. The details of this program have been reported previously [15]. Briefly, this telehealth program provides the following services: (1) biometric data, including single-lead electrocardiography, blood pressure, heart rate, and pulse oximetry, which are transferred from patients to our telehealth center daily and on-demand; (2) nurse case managers communicate with patients daily and on-demand by telephone to ensure compliance to medication and medical instruction; (3) full-time nurse case managers and cardiologists are in charge of telehealth care 24 hours a day; and (4) long-term medications and management are discussed with the patients’ primary care physicians if acute events occur. This telehealth program emphasizes compliance to medication and medical instruction, as well as prevention and early detection of clinical deterioration.

### Data Collection

All demographic and clinical data were obtained from the electronic database of the hospital. The diagnosis of a chronic disease was based on the electronic database. The discharge diagnosis was used if there was disagreement between outpatient and discharge diagnoses. The endpoints of this study were all-cause and cardiovascular hospitalizations.

**Figure 1:**
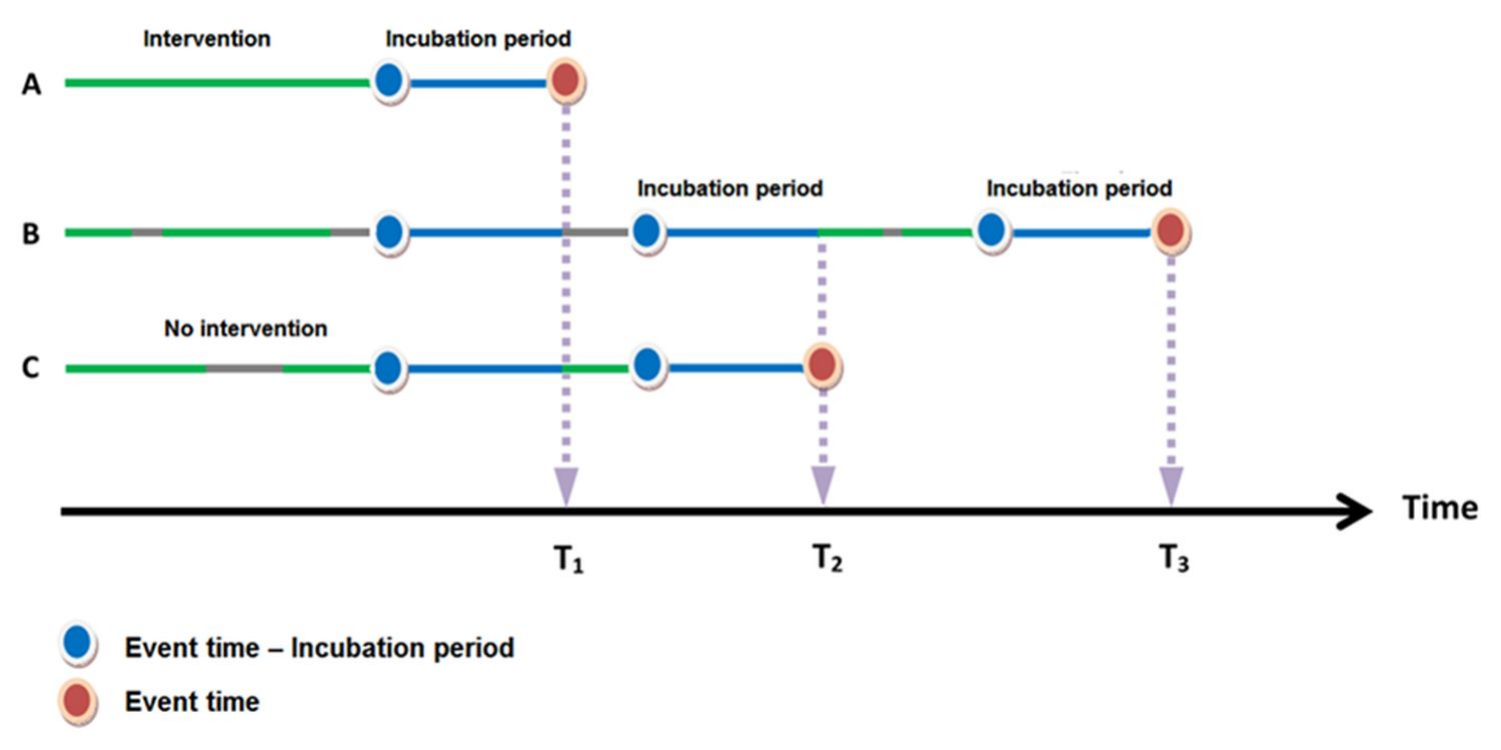
Incubation periods of the intervention’s effect at event times. The A, B, and C represent three fictitious participants. The green color indicates that the participant is using the telehealth service, the gray color indicates that the participant is not using telehealth service, and the blue color indicates that the participant is in the incubation period (defined as 28 days before a specific event). The T1, T2, and T3 are times when a participant develops an event (participants A, C, and B, respectively).

### Statistical Analysis

Statistical analysis was performed using the R 3.3.1 software (R Foundation for Statistical Computing, Vienna, Austria). In statistical testing, a two-sided *P* value ≤.05 was considered statistically significant. The distributional properties of continuous variables were expressed as mean and standard deviation, categorical variables were presented as frequency and percentage, and the survival outcome curves were estimated using the Kaplan-Meier method. In the univariate analysis, the differences in the distributions of continuous variables, categorical variables, and survival outcomes were examined among three groups of patients (normal renal function, CKD, and ESRD) using the Kruskal-Wallis test, chi-square test, Fisher exact test, or log-rank test as appropriate for the data type. Next, multivariate analysis was conducted by fitting the Cox proportional hazards models with time-dependent covariates (called the “Cox model” for simplicity) to estimate the adjusted effects of risk factors, prognostic factors, and predictors on survival outcomes.

### Contract Compliance Rate to the Telehealth Program

Contract compliance rate was defined as the percentage of days covered by the telehealth service duration within a certain time period before hospitalization. Specifically, we defined four time-varying variables of contract compliance for the time periods of 4, 8, 12, and 24 weeks, respectively, and then used them in fitting the Cox models. We assumed that the “incubation period” of the telehealth service was approximately 28 days; that is, the effect of the telehealth service might not appear until 28 days after signing or renewing the contract. The time-dependent contract compliance rates for 4, 8, 12, and 24 weeks were calculated using the following equation: contract compliance rates in *n* weeks = [(number of days covered by the telehealth program contract within *n* weeks before a specific event time – 28 days) / (7 days × *n*)] × 100%, where *n*=4, 8, 12, and 24. As shown in Figure 1, “a specific event time minus 28 days” is a specific event time moved 28 days backward to account for the assumed incubation period of the telehealth service.

We reorganized the original wide-form data into a long-form structure first, using the so-called “counting process style of input” for the survival outcome of interest, such as time to first hospitalization. Next, at each ordered event time of the survival outcome, we computed the values of the previously mentioned four time-dependent covariates for each of the patients at risk in the transformed long-form dataset. Finally, we fitted the Cox models to the long-form data with all relevant time-fixed and time-varying covariates using the coxph() function of the survival package in R.

To ensure the analysis quality, we used the model-fitting techniques for (1) variable selection, (2) goodness-of-fit assessment, and (3) regression diagnostics and remedies in our regression analyses. The stepwise variable selection procedure was applied to obtain the best candidate final regression model. The significance levels for entry and stay in the model were set to .15 to be conservative. The final regression model was identified manually by dropping the covariates with *P*>.05 one at a time until all regression coefficients were significantly different from zero.

Simple and multiple generalized additive models (GAMs) were fitted to detect nonlinear effects of continuous covariates and to identify appropriate cut-off point(s) for discretizing continuous covariates, if necessary, during the stepwise variable selection procedure. The vgam() function (with default values for the smoothing parameters) of the VGAM package was used to fit GAMs for continuous, binary, and count responses in R. Because GAMs were originally developed for smoothing the effects of continuous covariates in generalized linear models, we fitted GAMs of binary responses (eg, 1=hospitalization vs 0=no hospitalization) for our survival outcomes. In particular, we performed GAM analyses for the three groups of patients (normal, CKD, and ESRD) separately to detect heterogeneous nonlinear effects of continuous covariates in subgroups. Finally, the statistical tools of regression diagnostics for residual analysis, detection of influential cases, and check of multicollinearity were applied to discover any model or data problems. A variance-inflating factor value of 10 or greater in continuous covariates or 2.5 or greater in categorical covariates indicated the occurrence of multicollinearity problems among some of the covariates in the fitted regression model.

## Results

### Descriptive Statistics

In total, 715 patients were enrolled in this study and divided into three groups according to the status of their renal function: normal (n=490), with CKD (n=178), or with ESRD and on maintenance dialysis (n=47). The mean age was 66.6 (SD 15.0) years and 66.0% (472/715) of the participants were male. The patients’ baseline characteristics, stratified by renal function status, are shown in Table 1.

**Table 1:** Baseline demography of patients stratified according to renal function status (N=715).

Baseline characteristics		Normal renal function (n=490)	Chronic kidney disease (n=178)	End-stage renal disease on dialysis (n=47)	*P*
Age (years), mean (SD)		63.7 (14.8)	74.8 (11.7)	69.7 (11.8)	<.001
Gender (male), n (%)		333 (68.0)	112 (62.9)	27 (57.4)	.15
**Comorbidities, n (%)**					
	Hypertension	239 (48.7)	125 (70.2)	35 (74.5)	<.001
	Diabetes	125 (25.5)	92 (51.7)	32 (68.1)	<.001
	Cancer	57 (11.6)	20 (11.2)	4 (8.5)	.41
	Atrial fibrillation	94 (19.2)	41 (23.0)	4 (8.5)	.07
	Heart failure	130 (26.5)	80 (44.9)	30 (63.8)	<.001
	Myocardial infarction	84 (17.1)	26 (14.6)	7 (14.9)	.79
	Coronary artery disease	234 (47.8)	104 (58.4)	29 (61.7)	.07
	Peripheral arterial disease	16 (3.3)	24 (13.9)	8 (17.0)	<.001
	Cerebral vascular accident	50 (10.2)	32 (18.0)	6 (12.8)	.06
	Hemodialysis	0	0	35 (74.5)	<.001
	Peritoneal dialysis	0	0	12 (25.5)	<.001
**Medications, n (%)**					
	Angiotensin-converting-enzyme inhibitor	43 (8.8)	12 (6.7)	3 (6.4)	.69
	Angiotensin receptor blockers	197 (40.2)	84 (47.2)	17 (36.2)	.21
	Beta-blocker	278 (56.7)	91 (51.1)	25 (53.2)	.45
	Calcium channel blocker	149 (30.4)	87 (48.9)	22 (46.8)	<.001
	Metformin	44 (9.0)	18 (10.1)	0	.12
	Sulfonylurea	47 (9.6)	36 (20.2)	4 (8.5)	.001
	Glitazones	3 (0.6)	6 (3.3)	0	.06
	Dipeptidyl peptidase-4 inhibitor	41 (8.4)	33 (18.5)	12 (25.5)	<.001
	Insulin	3 (0.6)	12 (6.7)	5 (10.6)	<.001
	Spironolactone	55 (11.2)	34 (19.1)	0	<.001
	Thiazide	42 (8.6)	24 (13.5)	2 (4.3)	.08
	Statins	179 (36.5)	70 (39.3)	14 (29.8)	.69
	Fenofibrate	5 (1.0)	17 (9.6)	2 (4.3)	<.001
Telehealth contract duration (days), mean (SD)		297 (410)	436 (506)	345 (407)	.001

**Figure 2:**
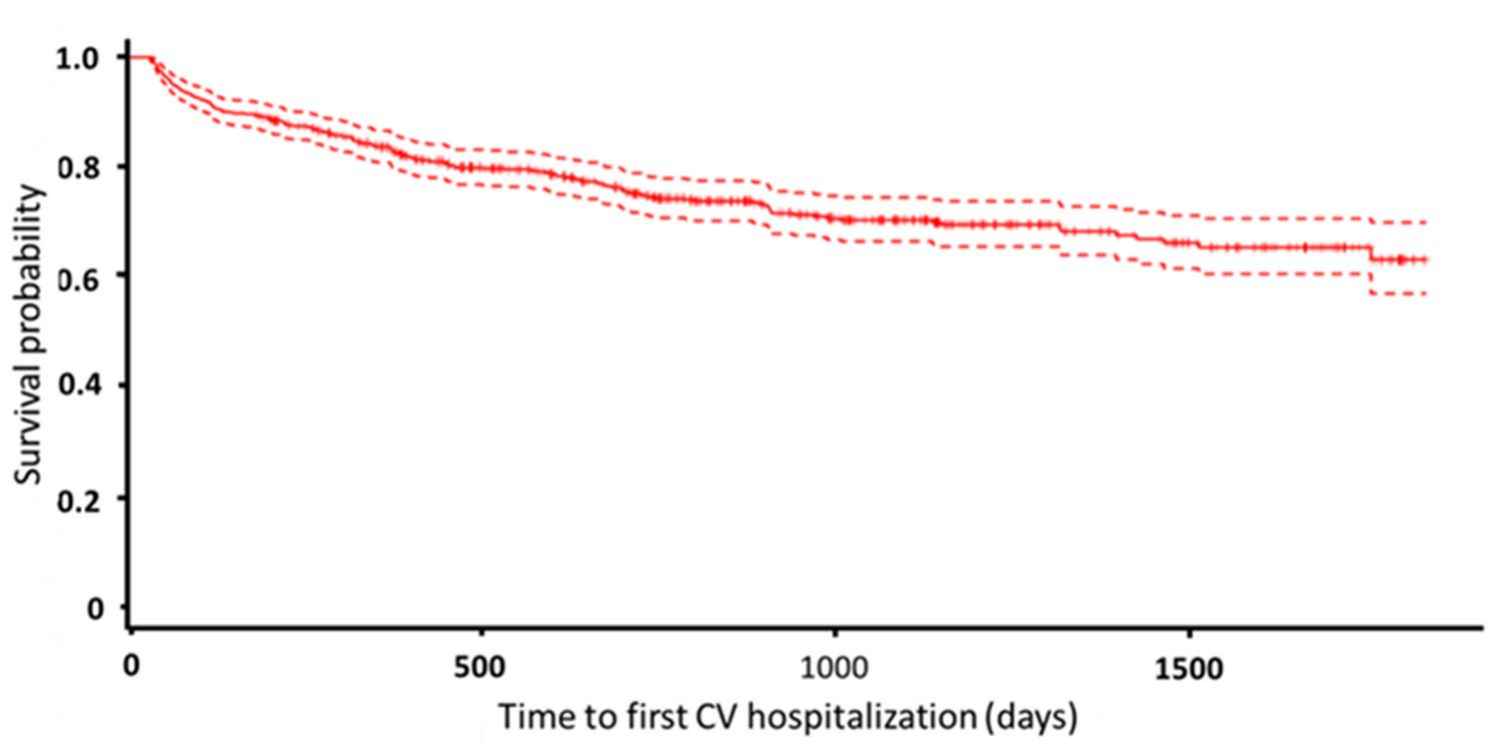
Kaplan-Meier curve for time to the first cardiovascular (CV) hospitalization.

**Figure 3:**
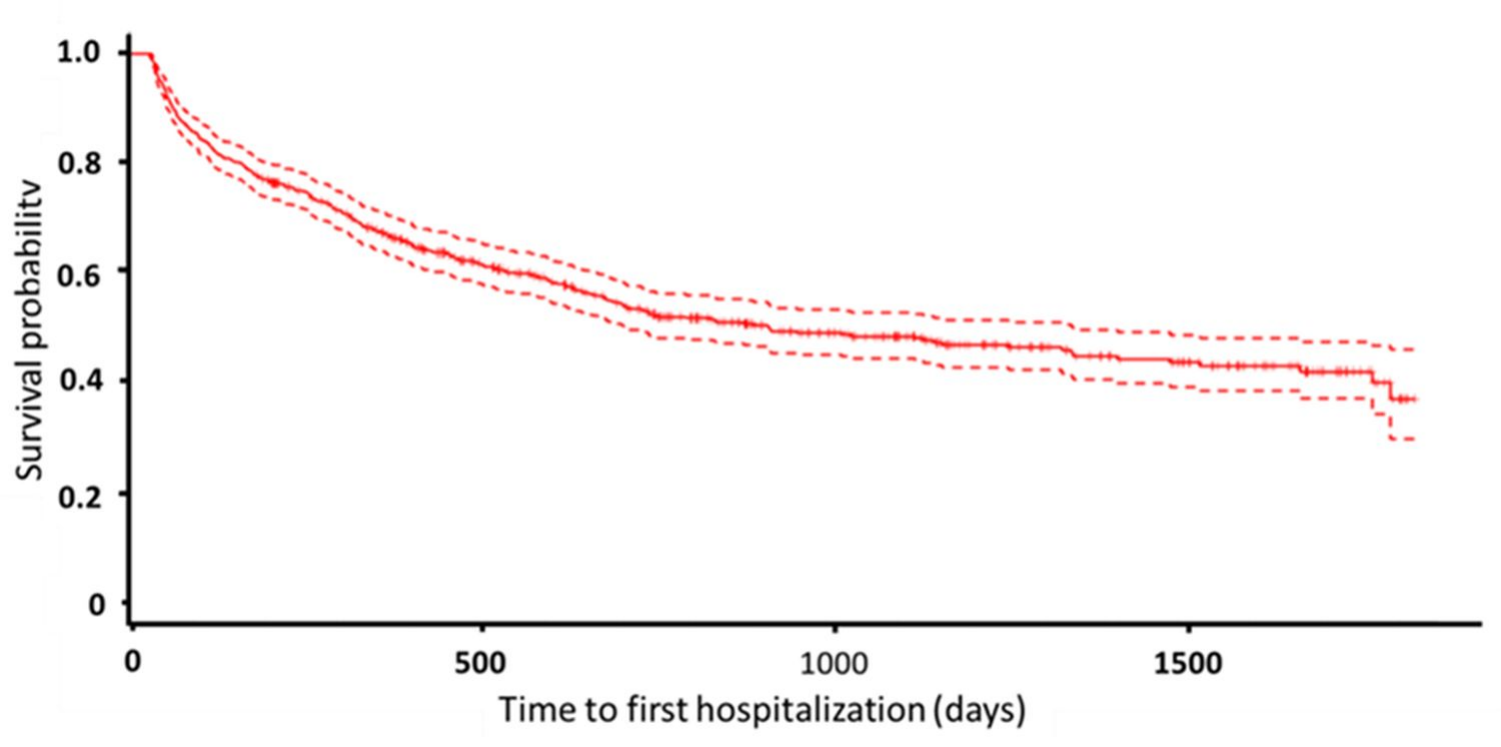
Kaplan-Meier curve for time to the first all-cause hospitalization.

The three groups were significantly different in terms of age and the proportion with hypertension, diabetes, heart failure, or peripheral artery disease, and the proportion receiving dialysis. The duration of telehealth use was different between the three study groups (longest in the group with CKD compared with the group with normal renal function and the group with ESRD on dialysis; Table 1). The median follow-up time was 694 days (IQR 338-1163). Because of the different follow-up times, events were divided according to follow-up time (days) in the subsequent analysis. During the follow-up period, there were 57 emergency room visits, 173 cardiovascular hospitalizations, and 350 all-cause hospitalizations. The Kaplan-Meier curves for time to first cardiovascular and all-cause hospitalization are shown in Figures 2 and 3, respectively.

**Table 2:** Univariate analysis of first cardiovascular hospital admission according to renal function status.

Variable	All patients		Normal renal function		Chronic kidney disease		End-stage renal disease on dialysis	
	OR (95% CI)	*P* value	OR (95% CI)	*P* value	OR (95% CI)	*P* value	OR (95% CI)	*P* value
Male	1.14 (0.78-1.7)	.51	1.39 (0.82-2.42)	.22	0.93 (0.46-1.92)	.87	1.61 (0.40-6.73)	.54
Hypertension	0.93 (0.64-1.34)	.72	0.69 (0.42-1.12)	.13	0.74 (0.35-1.57)	.47	1.79 (0.34-10.5)	.48
Diabetes	1.74 (1.2-2.54)	.002	1.50 (0.88-2.53)	.11	1.15 (0.58-2.3)	.74	1.70 (0.36-8.57)	.51
Cancer	0.53 (0.26-1.01)	.05	0.61 (0.24-1.36)	.29	0.30 (0.05-1.10)	.08	0 (0.18-∞)	.49
Atrial fibrillation	1.06 (0.67-1.67)	.82	1.45 (0.80-2.56)	.18	0.61 (0.24-1.44)	.25	1.97 (0.09-124)	>.99
Heart failure	2.08 (1.43-3.03)	<.001	1.84 (1.09-3.08)	.02	1.61 (0.80-3.25)	.18	1.65 (0.38-7.56)	.52
Myocardial infarction	1.43 (0.89-2.27)	.12	1.58 (0.86-2.82)	.13	1.32 (0.49-3.42)	.65	1.32 (0.19-10.44)	>.99
Coronary artery disease	1.42 (0.99-2.06)	.05	1.59 (0.98-2.59)	.05	0.99 (0.49-1.99)	>.99	1.08 (0.26-4.54)	>.99
Peripheral artery disease	2.70 (1.36-5.34)	.003	2.36 (0.59-8.42)	.16	1.37 (0.48-3.75)	.63	7.27 (0.76-366.79)	.09
Cerebral vascular accident	1.15 (0.65-1.98)	.59	1.57 (0.72-3.24)	.25	0.72 (0.25-1.86)	.52	0.43 (0.03-3.46)	.41
Hemodialysis	3.13 (1.37-7.18)	.004	N/A	N/A	N/A	N/A	1.07 (0.23-5.09)	>.99
Peritoneal dialysis	2.82 (0.74-10.69)	.09	N/A	N/A	N/A	N/A	0.93 (0.20-4.44)	>.99
Angiotensin-converting-enzyme inhibitor	1.86 (1.00-3.42)	.03	2.14 (0.99-4.48)	.04	1.63 (0.37-6.75)	.51	1.97 (0.09-124)	>.99
Angiotensin receptor blockers	0.94 (0.65-1.36)	.79	1.07 (0.66-1.74)	.81	0.78 (0.39-1.57)	.51	0.75 (0.16-3.38)	.74
Beta-blocker	1.37 (0.94-1.98)	.94	1.47 (0.90-2.43)	.13	1.19 (0.60-2.39)	.63	1.97 (0.48-8.53)	.35
Calcium channel blocker	0.98 (0.67-1.43)	.67	0.69 (0.39-1.18)	.17	0.93 (0.47-1.87)	.87	1.61 (0.40-6.67)	.54
Metformin	2.1 (1.11-3.86)	.01	3.23 (1.53-6.72)	.002	1.06 (0.26-3.73)	>.99	N/A	N/A
Sulfonylurea	1.2 (0.65-2.02)	.58	1.11 (0.45-2.52)	.83	1.99 (0.40-2.37)	>.99	0.95 (0.06-14.39)	>.99
Glitazones	0.92 (0.09-5.2)	>.99	1.84 (0.03-35.75)	.51	0.47 (0.01-4.87)	.66	N/A	N/A
Insulin	0.98 (0.27-2.95)	>.99	0 (0-8.93)	>.99	0.61 (0.10-2.59)	.55	0.95 (0.06-14.39)	>.99
Spironolactone	2.13 (1.28-3.52)	.002	2.23 (1.12-4.34)	.02	2.23 (0.94-5.29)	.06	N/A	N/A
Thiazide	1.72 (0.95-3.06)	.94	1.87 (0.82-4.06)	.10	1.55 (0.56-4.16)	.35	0.95 (0.01-78.40)	>.99
Statins	1.52 (1.05-2.19)	.02	1.49 (0.91-.2.43)	.09	01.37 (0.35-1.48)	.40	0 (0.21-4.10)	>.99
Fenofibrate	1.16 (0.79-1.67)	.46	0 (0-4.01)	.59	0.73 (0.24-3.22)	.40	0 (0.18-∞)	.49

### Univariate Analysis for First Cardiovascular Hospitalization

We performed a univariate analysis using each variable associated with the first cardiovascular hospitalization (Table 2). Among all study participants, a history of diabetes (OR 1.74, 95% CI 1.20-2.54, *P*=.002), heart failure (OR 2.08, 95% CI 1.43-3.03, *P*<.001), peripheral artery disease (OR 2.7, 95% CI 1.36-5.34, *P=*.003), or hemodialysis (OR 3.13, 95% CI 1.37-7.18, *P=*.004) were associated with a higher risk of first cardiovascular hospitalization. The use of angiotensin-converting-enzyme inhibitors (OR 1.86, 95% CI 1.00-3.42, *P*=.03), metformin (OR 2.10, 95% CI 1.11-3.86, *P=*.01), spironolactone (OR 2.13, 95% CI 1.28-3.52, *P=*.002), or statin (OR 1.52, 95% CI 1.05-2.19, *P=*.02) were also significantly associated with the first cardiovascular hospitalization. Among patients with normal renal function, only heart failure (OR 1.84, 95% CI 1.09-3.08, *P=*.02) was associated with a higher risk of first cardiovascular hospitalization. The associations between medication use and hospitalization in patients with normal renal function were similar to the overall population, except in the case of statins. However, there was no association between clinical factors or the use of medication and the first hospitalization in patients with CKD or ESRD.

### Hazard Ratio for First Cardiovascular and All-Cause Hospitalization

**Figure 4:**
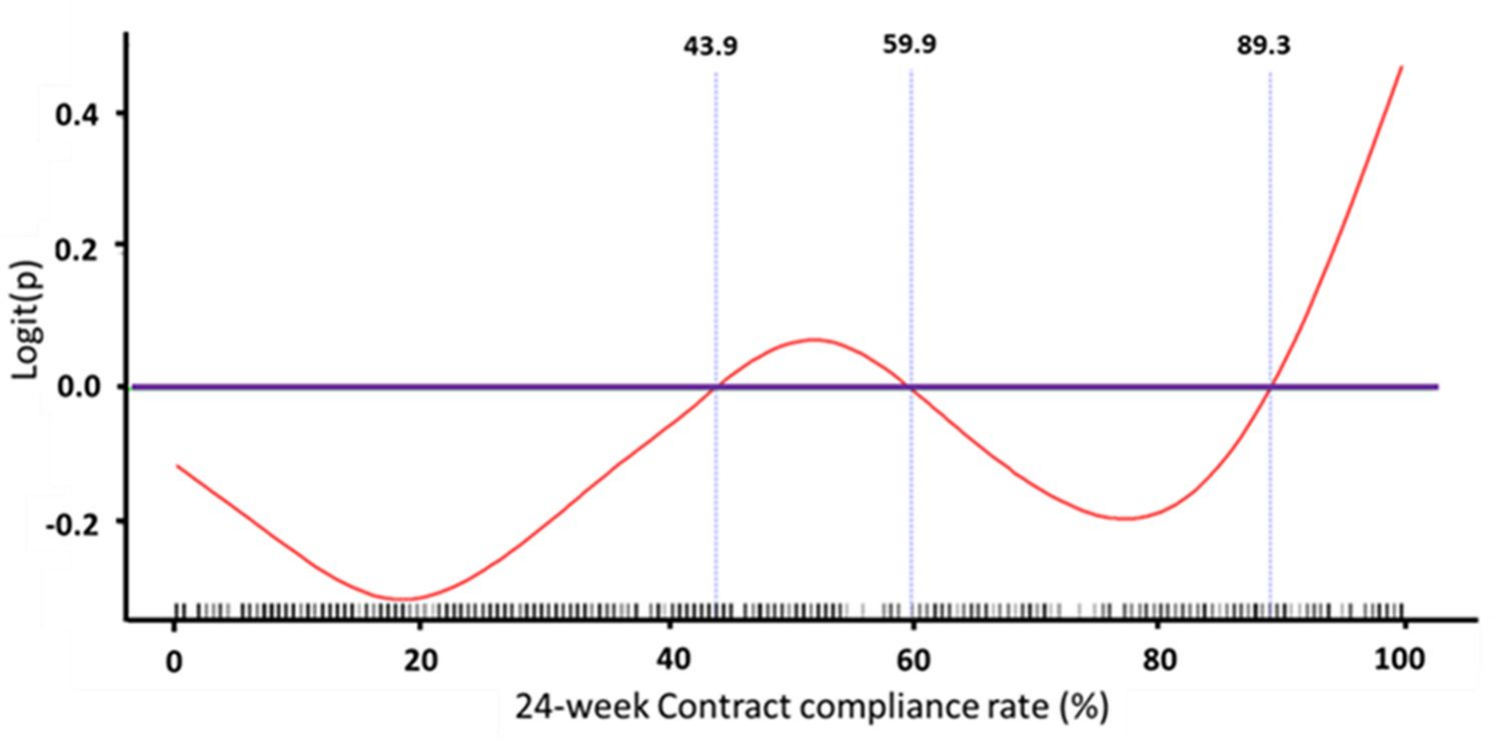
Generalized additive model (GAM) plot to assess the nonlinear relationship between contract compliance rate within 24 weeks and risk of cardiovascular hospitalization. Note: logit(P) was the logit transformation of probability(P)=ln(P/[1–P]).

We used Cox proportional hazards models with time-dependent covariates (ie, the Cox model) to estimate the effect of predictors on first cardiovascular hospitalization (Table 3). The results showed that previous emergency department admission, peripheral arterial disease, the use of spironolactone, the use of statins or metformin in patients with normal renal function, the presence of atrial fibrillation in patients with normal renal function, and the presence of ESRD were associated with a higher risk of first cardiovascular hospital admission. In addition, contract compliance rate of the telehealth program among patients with CKD was also associated with a higher risk of first cardiovascular hospital admission. We used a GAM plot to assess the nonlinear relationship between contract compliance rate within 24 weeks and risk of cardiovascular hospitalization (Figure 4). The results showed that there was a triphasic, or U-shaped, relationship: patients with very low or very high contract compliance rates within 24 weeks were associated with a higher risk of cardiovascular hospitalization.

**Figure 5:**
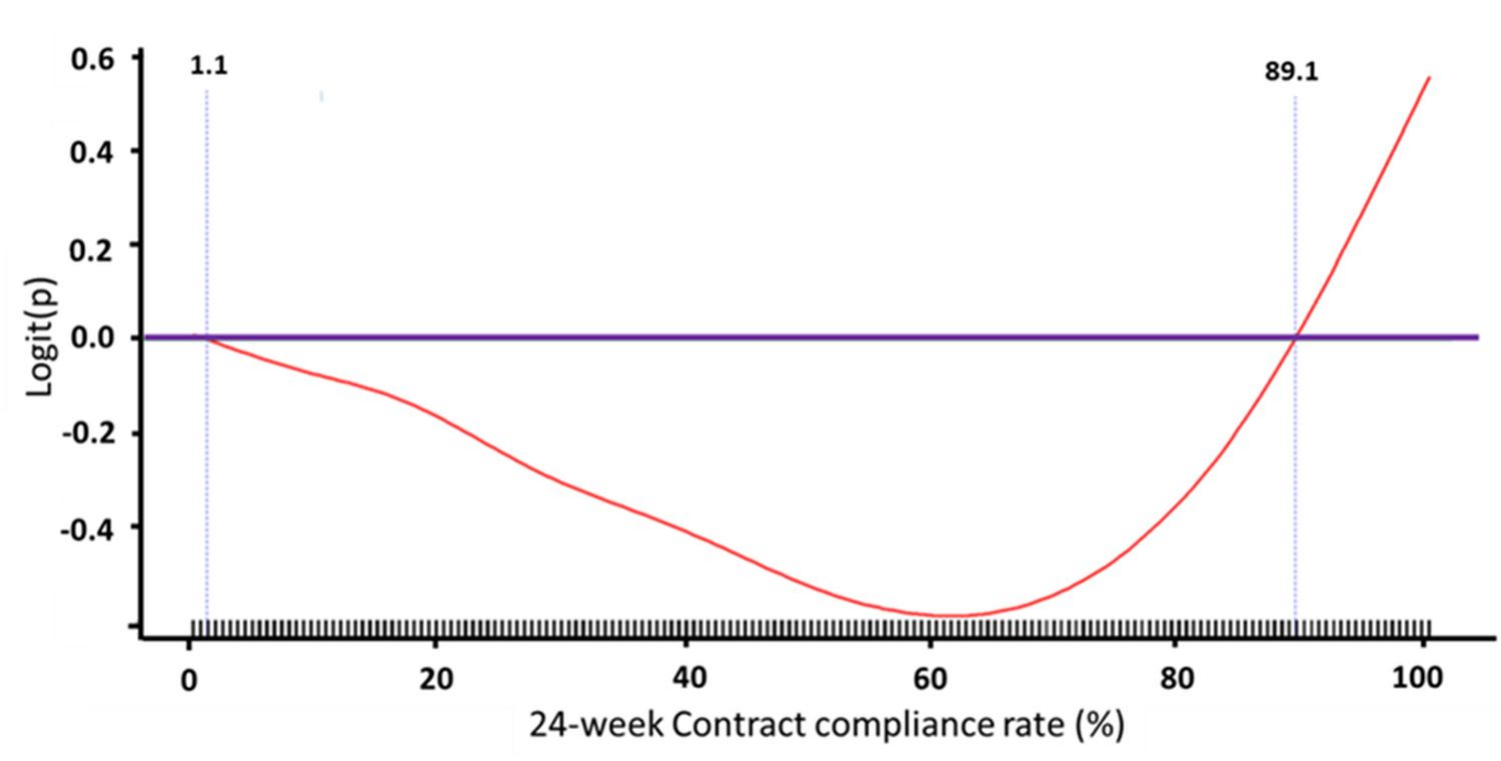
Generalized additive model (GAM) plot to assess the nonlinear relationship between contract compliance rate within 24 weeks and risk of cardiovascular hospitalization for participants with normal renal function. Note: logit(P) was the logit transformation of probability(P)=ln(P/[1–P]).

**Figure 6:**
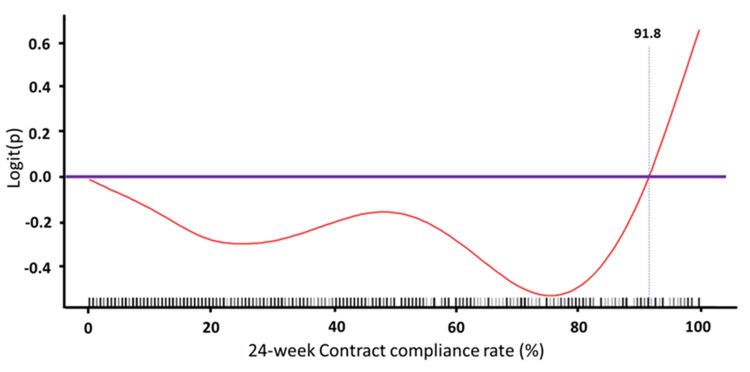
Generalized additive model (GAM) plot to assess the nonlinear relationship between contract compliance rate within 24 weeks and risk of cardiovascular hospitalization for participants with chronic kidney disease. Note: logit(P) was the logit transformation of probability(P)=ln(P/[1–P]).

**Figure 7:**
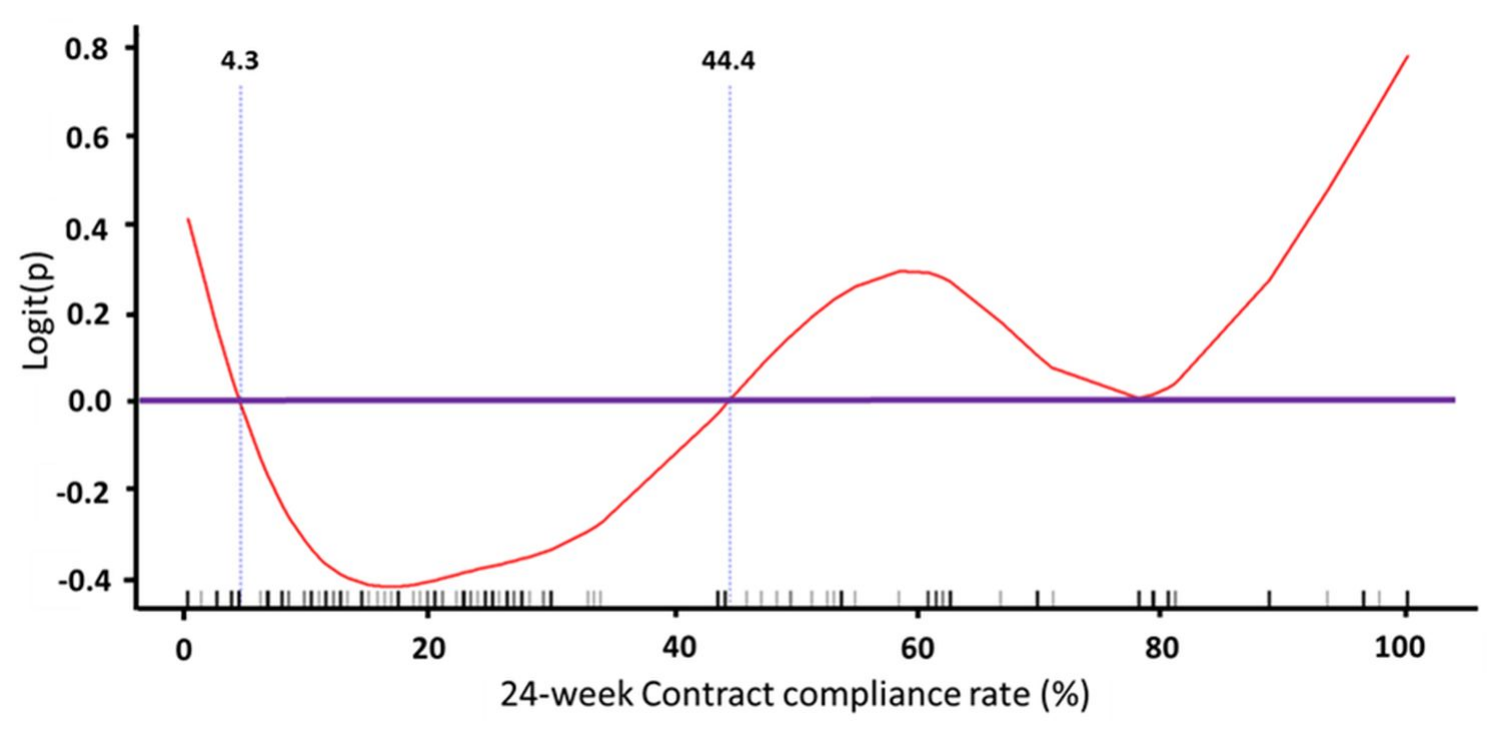
Generalized additive model (GAM) plot to assess the nonlinear relationship between contract compliance rate within 24 weeks and risk of cardiovascular hospitalization for participants with end-stage renal disease. Note: logit(P) was the logit transformation of probability(P)=ln(P/[1–P]).

The Cox model for estimating the effects of predictors on the first all-cause hospitalization yielded similar results (Table 4). The presence of cancer and the use of spironolactone were associated with a higher risk of first all-cause hospitalization, whereas the presence of normal renal function and the use of fenofibrate were associated with a lower risk of first all-cause hospitalization. We used a GAM plot to assess the nonlinear relationship between contract compliance rate within 24 weeks and risk of all-cause hospitalization. The results showed that there was a triphasic relationship: patients with very low and very high contract compliance rates within 24 weeks were associated with a higher risk of cardiovascular hospitalization in all three patient groups (normal renal function, CKD, and ESRD; Figures 5-7). Notably, patients with ESRD had a lower threshold for the right arm of the U-shaped relationship compared with the other two groups; in other words, their risk of all-cause hospitalization was higher if their contract compliance rate was greater than 44.4% compared with 89.1% and 91.8% for patients with normal renal function and CKD, respectively.

**Table 3:** Hazard ratio for first cardiovascular hospitalization.

Variable	Hazard ratio (95% CI)	*P* value
Pre-emergency department admission	7.6 (5.5-10.7)	<.001
Peripheral arterial disease	2.0 (1.3-2.7)	.006
Spironolactone	2.1 (1.7-8.5)	<.001
Normal renal function × statins	1.8 (1.4-3.2)	.002
Normal renal function × metformin	2.1 (1.2-3.5)	.007
Normal renal function × atrial fibrillation	1.6 (1.0-2.6)	.03
Chronic renal disease × 24-week contract compliance	2.5 (1.6-4.1)	<.001
End-stage renal disease	4.1 (2.5-6.9)	<.001

**Table 4:** Hazard ratio for first all-cause hospitalization.

Variable	Hazard ratio (95% CI)	*P*
Normal renal function	0.4 (0.3-0.5)	<.001
Cancer	1.5 (1.1-2.0)	.02
Fenofibrate	0.4 (0.2-0.9)	.03
Spironolactone	2.2 (1.6-2.9)	<.001
Normal renal function × 24-week contract compliance	2.2 (1.5-3.2)	<.001
Normal renal function × diabetes	1.6 (1.1-2.1)	.007
Normal renal function × cerebral vascular accident	1.7 (1.1-2.6)	.02
Normal renal function × peripheral arterial disease	2.1 (1.1-3.8)	.02
Chronic renal disease × 24-week contract compliance	2.0 (1.3-3.2)	.002

## Discussion

### Major Findings

The major findings of this study were as follows: (1) higher contract compliance rate with the telehealth program was associated with a higher risk of first cardiovascular or all-cause hospitalization; (2) for patients receiving the telehealth program, the level of renal function was associated with hospitalization and patients with ESRD were associated with a higher risk of first cardiovascular or all-cause hospitalization after adjusting for comorbidities and medications; and (3) there was an interaction between the effect of contract compliance rate and renal function on the time to first hospitalization. Patients with ESRD had a higher risk of hospitalization at a lower contract compliance rate, compared with patients with CKD or normal renal function.

In our previous study, patients receiving the telehealth program were associated with lower all-cause mortality than those receiving usual care [5]. Based on that study and these findings, we suggest that one reason for this was timely hospitalization for disease management. Patients receiving the telehealth program and exhibiting high contract compliance rate may be more vulnerable because of complex underlying diseases and comorbidity, and if so would require comprehensive care, with which the telehealth program would assist. Our results highlight the effect of contract compliance rate in patients receiving the benefits of the telehealth program.

### Contract Compliance Rate and Hospitalization

The analyses of contract compliance rate and risk of hospitalization revealed a triphasic relationship. Patients with a very low contract compliance rate were associated with a higher risk of hospitalization. This implies that duration of contract to the telehealth program of close to or less than 28 days has no effect on the prevention of acute deterioration in disease. Patients with a midrange contract compliance rate were associated with a lower risk of hospitalization. This highlights the usefulness of the telehealth program for most patients. Unexpectedly, patients with higher contract compliance were associated with a higher risk of hospitalization. This last finding seems counterintuitive because most compliance studies show that higher compliance to pharmacotherapy is associated with a lower risk of complications [16-19]. The issue of compliance, however, has not been formally explored in telehealth studies and should not be taken for granted [20]. The effect of compliance to a telehealth program is difficult, if not impossible, to evaluate using a randomized controlled trial. Therefore, careful analysis of observational data is needed to explore this issue. In randomized controlled trials, reported compliance is usually good, with up to 85% of participants still using the telehealth program until the end of the study [21,22]. However, the impact of contract compliance rate on the risk for hospitalization in the real world has not been reported. Our report shows that with a low to midrange contract compliance rate, a higher contract compliance rate is associated with a lower risk of hospitalization.

A similar U-shaped curve (the “three-phase terrain”) of hospital admission has been observed in lifetime analysis of heart failure [23,24]. In that report, 30% of all cardiovascular readmissions occurred within the first 2 months of hospital discharge, and 50% occurred within the 2 months before death, with much lower admission rates (15%-20%) observed in the intercurrent plateau phase [23]. To achieve a longer plateau phase, patient care should extend into the home to monitor and maintain stability, and into active intervention for ambulatory patients who have early signs of organ decompensation [25]. Given the predictive or preventive scenarios for heart failure admission, the relationship between high admission risk and compliance with telehealth demonstrated in this study merits further consideration. There are two possible explanations for this result. First, we consider the predictive scenario [25]. Because participation in the telehealth program is voluntary and self-funded, contract compliance rate is largely determined by the needs of the disease process and the sense that these needs have been met as a result of participation in the program. Therefore, patients with greater disease severity remain in the telehealth program for longer. However, greater disease severity is also associated with an increased risk of hospitalization. Second, to prevent further deterioration of the clinical situation, the telehealth program might partly increase the rate of hospitalization if a decline in organ function cannot be managed in outpatient or emergency departments. We have shown in our previous study that our program is associated with lower all-cause mortality rates. According to the preventive scenario, the program increases the rate of hospitalization so that crises can be managed earlier, rapid deterioration can be halted, and overall outcomes can be improved.

### Renal Function and Hospitalization

Telehealth programs in CKD [26,27] have been less well studied compared with other chronic conditions such as heart failure, chronic obstructive pulmonary disease, or diabetes [2,28-31]. Knowledge about the efficacy and cost-effectiveness of telehealth among patients with CKD is still lacking. In a recent study, researchers showed that a telehealth program provided by an interprofessional team is a feasible approach for this patient group, but that it did not result in less hospitalizations, fewer emergency department visits, or lower all-cause mortality [26]. In our previous report, we showed that CKD was a predictor for all-cause mortality among these patients [5]. These results show that among patients receiving the telehealth program, renal function status was still a predictor for first hospitalization. Our program is specialized for risk factor control and adherence to recommended management guidelines among these patients. Determining whether telehealth improves the outcome of these patients requires further effectiveness comparison studies.

### Interaction Between Renal Status and Contract Compliance Rate With Telehealth

We observed a significant interaction between the effects of renal status and contract compliance rate on hospitalization. Among patients with normal renal function and CKD, a contract compliance rate greater than 90% was associated with a higher rate of all-cause hospitalization, whereas among patients with ESRD, a contract compliance rate greater than 44% was associated with a higher rate of hospitalization. It is possible that contract compliance rate is a reflection of patient’s awareness about their disease severity. The significantly lower contract compliance rate associated with increased hospitalization rate in patients with ESRD might suggest that, among ESRD patients, their awareness may be lower than their real need for medical help. This could be due to factors such as low awareness of the severity and long-term complications of their disease, low awareness of the importance of compliance to recommended management guidelines, or accessibility of the health care system via alternative care providers (eg, the dialysis center). Whatever the case, noncompliance to recommended management guidelines in this patient group is associated with a higher risk of long-term complications [32,33]. From the perspective of telehealth care providers, it would be prudent to ascertain that patients with ESRD exhibit good compliance to standard management. Renal status, therefore, is a major determinant for risk evaluation of patients receiving telehealth care programs.

### Limitations

There are several limitations in our study. Firstly, this is not a randomized controlled study comparing patients with and without access to the fourth-generation telehealth care program. Therefore, causation cannot be determined from our results. Secondly, there were fewer patients with CKD and ESRD (especially the latter) than with normal renal function. The effect of contract compliance rate was not statically significant for patients with ESRD, which may be due to limited patient numbers. Thirdly, our analysis did not account for recurrent events. It may be important to differentiate patients who are more likely to undergo several admissions from those with only one admission. Finally, our total patient number was limited. We have only listed the factors that were significant for predicting the first cardiovascular hospital admission in our final model; some predictors in the initial analyses were not statistically significant in the more comprehensive analyses (when the patients were divided into three groups). Thus, the interaction between clinical factors and each group may not be clear due to our limited patient numbers.

### Conclusion

In summary, we found that contract compliance rate to the fourth-generation telehealth program is associated with cardiovascular outcomes, especially in patients with CKD. We demonstrated a triphasic relationship between contract compliance rate and risk of hospitalization among patients with CKD. Patients with low and high contract compliance rate to the telehealth program were associated with higher risk for hospitalization. A large, prospective, randomized controlled study is needed in these high-risk CKD patients to determine whether the telehealth program is beneficial.

## Abbreviations

CKD: chronic kidney disease
CVD: cardiovascular disease
ESRD: end-stage renal disease
GAM: generalized additive model
GFR: glomerular filtration rate
